# Compartment Syndrome Secondary to Knee Lipohemarthrosis

**DOI:** 10.7759/cureus.16946

**Published:** 2021-08-06

**Authors:** Madeleine E Kim, Thor S Stead, Latha Ganti

**Affiliations:** 1 Emergency Medicine, Baylor School, Chattanooga, USA; 2 Epidemiology and Public Health, Alpert Medical School of Brown University, Providence, USA; 3 Emergency Medicine, Envision Physician Services, Plantation, USA; 4 Emergency Medicine, University of Central Florida College of Medicine, Orlando, USA; 5 Emergency Medicine, Ocala Regional Medical Center, Ocala, USA; 6 Emergency Medicine, HCA Healthcare Graduate Medical Education Consortium Emergency Medicine Residency Program of Greater Orlando, Orlando, USA

**Keywords:** lipohemarthrosis, tibial fracture, compartment syndrome, fat-fluid level, fibular fracture, intra-articular fracture, compartment pressure

## Abstract

When treating patients presenting with knee trauma or intra-articular fracture, clinicians should maintain a high index of suspicion for lipohemarthrosis. Diagnosis of lipohemarthrosis can be accomplished via visualization of a fat-fluid level. Increased fluid and pressure build-up within the joint space may lead to compartment syndrome, which requires emergency compartment fasciotomy. In this paper, we discuss the importance of identifying lipohemarthrosis in patients presenting with intra-articular fracture, as well as the necessity of frequent patient re-evaluations in order to monitor the onset of compartment syndrome.

## Introduction

Knee traumas account for over 500,000 visits to the emergency department (ED) per year in the United States [1-3]. When displaced, intra-articular fractures may result in bone marrow and blood spilling into the surrounding joint capsule [4]. This is defined as lipohemarthrosis. Lipohemarthrosis is not entirely uncommon in intra-articular fractures, occurring in 35%-41% of cases [1,5,6]. In the knee, lipohemarthrosis is most likely to accompany tibial plateau fractures and may require orthopedic surgery in certain cases [7]. Lipohemarthrosis can be diagnosed with plain radiography (X-rays) or computed tomography (CT) of the injured knee, with axial CT images oftentimes allowing visualization of fat-fluid levels within the joint [1]. When intra-articular fractures are displaced and bone marrow and blood spill into the joint, the differing densities of the bone marrow and blood cause separation of fat and fluid, forming the fat-fluid levels visible in scans. Over time, the fluid can further separate into serum and red blood cell precipitate layers, forming three layers referred to as double fluid-fluid levels [1]. Build-up of fluid within a joint may lead to compartment syndrome, a limb-threatening complication [8]. Thus, it is necessary for clinicians to have a high index of suspicion for lipohemarthrosis and therefore compartment syndrome when treating patients with knee trauma. In this paper, we discuss a case in which a patient presented with an intra-articular tibial fracture and a right proximal fibular fracture, as well as signs and symptoms of early compartment syndrome due to lipohemarthrosis in subsequent re-evaluations.

## Case presentation

A 30-year-old male presented to the ED via emergency medical services (EMS) with right knee pain and swelling after sustaining a fall. The patient stated that he was standing on a conveyor belt on his left lower extremity and then fell, landing with his right knee in varus stress. The fall happened from approximately 3 feet, and upon falling, he felt an immediate pop, accompanied by intense pain that radiated distally down to his foot. The patient reported the severity of pain at onset as 10/10 and was unable to bear weight on his right lower extremity. En route, EMS reported that the patient received 100 mcg of intravenous (IV) fentanyl, with improvement in pain from 10/10 to 2/10, as well as a 500 cc bolus of IV saline. 

The patient had a temperature of 36.5°C, with a blood pressure of 135/85 mmHg and a pulse of 53 beats per minute. Electrocardiogram (ECG) showed slight sinus bradycardia but the patient was asymptomatic. Oxygen saturation was 95% on room air, and respiratory rate was 14 breaths per minute. Though otherwise healthy, the patient did admit to smoking a pack of cigarettes every day. Physical examination showed no abnormalities outside of the affected extremity. Examination of the right lower extremity showed moderate swelling over the right anterior knee with moderate tenderness to palpation over the right anterior and right lateral knee. There was moderate tenderness to the right proximal fibula, but the anterior compartments were soft, and skin was intact. The patient’s calf was not tender. His right foot was diffusely tender to palpation, but otherwise had no swelling, erythema, or ecchymosis. The patient was able to move all digits and his dorsalis pedis and posterior tibial pulses were equal bilaterally. On admission, the patient was agitated but otherwise alert and oriented.

**Figure 1:**
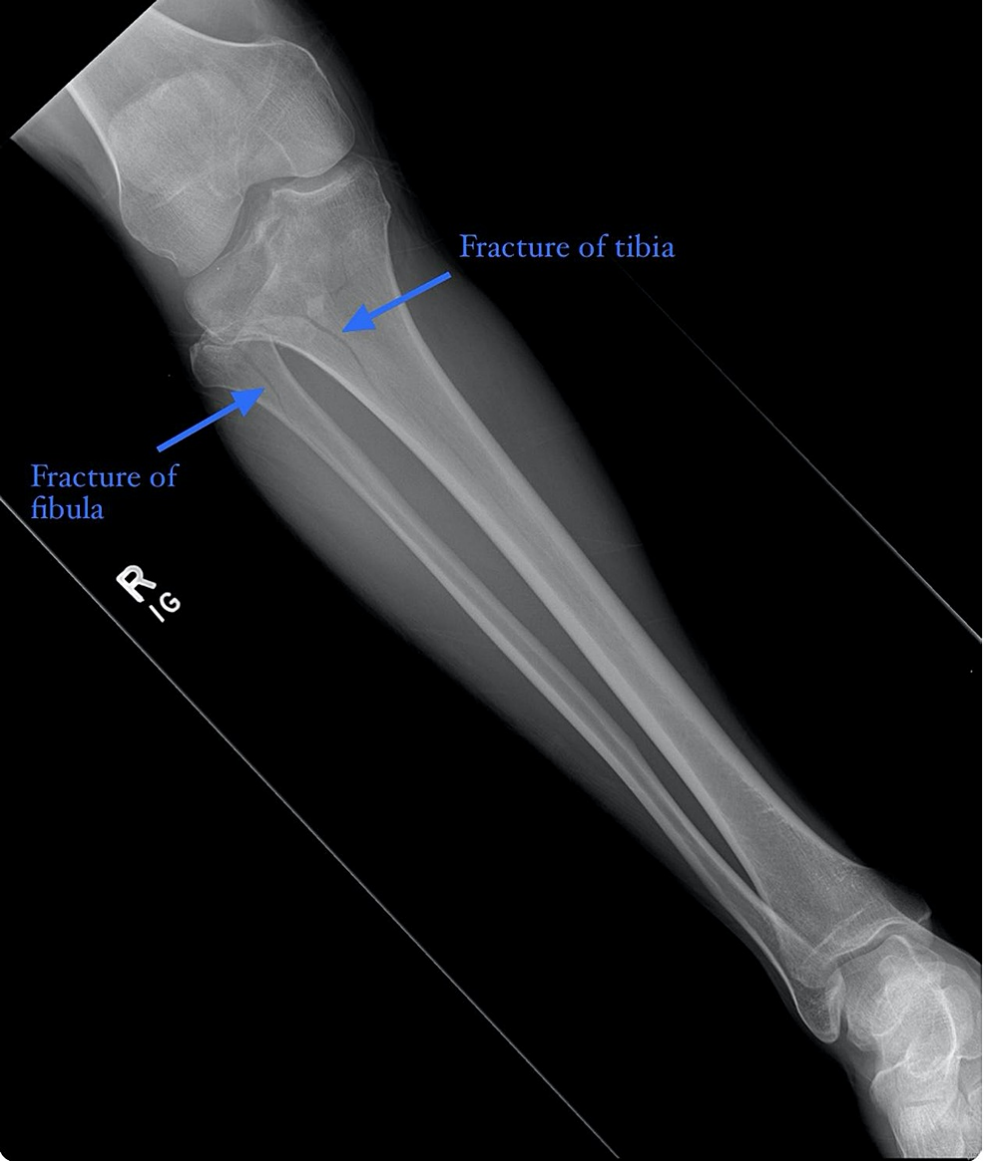
Plain radiograph of the right tibia fibula showed comminuted proximal tibial and fibular fractures (blue arrows) with evidence of intra-articular extension, right knee joint effusion, and lipohemarthrosis

X-ray of the right femur showed right tibial and fibular fractures without femoral fracture. X-ray of the right foot showed no evidence of acute fracture or dislocation. X-ray of the right tibia fibula showed comminuted proximal tibial and fibular fractures with evidence of intra-articular extension, right knee joint effusion, and lipohemarthrosis (Figure 1).

**Figure 2:**
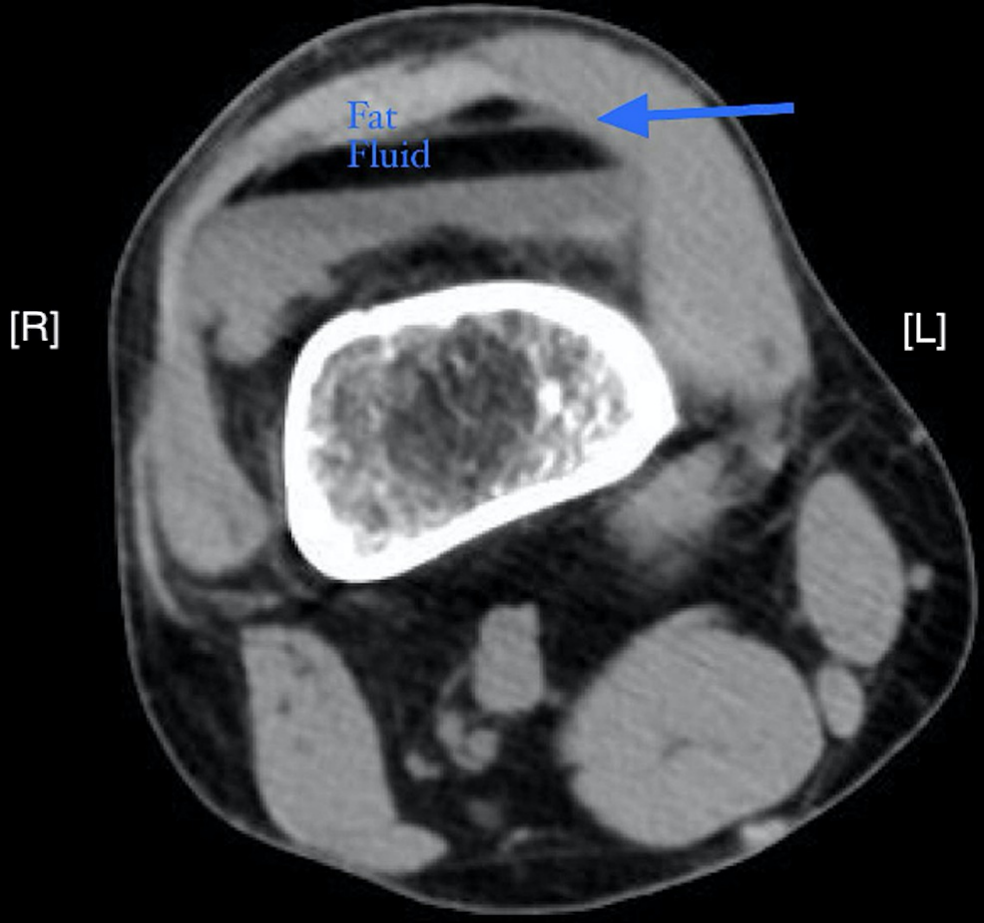
Axial CT image without contrast of the right lower extremity showed the fat-fluid level (blue arrow) indicative of lipohemarthrosis. Along with moderate lipohemarthrosis, soft tissue edema was identified. CT also revealed evidence of complex comminuted depressed fracture of the proximal tibia, involving mainly the lateral plateau. There is also a fracture of the fibular head

A CT of the lower extremity without contrast yielded evidence of a complex comminuted depressed fracture of the proximal tibia, involving mainly the lateral plateau, as well as a fracture of the fibular head. Moderate lipohemarthrosis and soft tissue edema was also identified (Figure 2). 

The patient was diagnosed with a right proximal intra-articular tibial fracture (tibial plateau fracture), right knee effusion, right proximal fibular fracture, and lipohemarthrosis. Re-evaluations of the patient at first revealed no signs or symptoms of compartment syndrome, but by the fourth re-evaluation, the patient presented with anterior compartments of the right lower extremity warm and firm to touch. The extremity was placed in an increased level of elevation and was iced, and a CT without contrast was ordered following observed signs of early compartment syndrome. At the time of the fourth re-evaluation, the patient’s compartment pressure was measured at 62 mmHg. ED staff continued to assess the compartment pressures of the patient with a commercially available manometer (Table 1). 

**Table 1:** Table delineating re-evaluations of the patient including time compartment pressure recorded at each re-evaluation pt: patient; NVIT: neurovascularly intact; RLE: right lower extremity; DP & PT: dorsalis pedalis & posterior tibial

Patient Re-Evaluation #	Time	Patient Notes
Re-Evaluation 1	14:20	Pt in mild distress, secondary to pain, which is currently 4/10. Pt remains NVIT with no signs or symptoms of compartment syndrome.
Re-Evaluation 2	14:54	Pt pain controlled, remains NVIT distally. Anterior compartments remain soft, showing no signs or symptoms of compartment syndrome.
Re-Evaluation 3	15:38	Pt awake/alert complaining of 7/10 pain, requesting more analgesia. Morphine ordered (4 mg at this time).
Re-Evaluation 4	16:00	Pt in moderate distress secondary to pain. Morphine 4 mg administered. Anterior compartments of RLE are warm and firm to touch. Capillary refill < 2 seconds, sensation intact distally, DP & PT pulses present and equal bilaterally. RLE elevated and iced. Compartment pressure was measured at 62 mmHg. Will continue to assess compartment pressures via Stryker needle due to signs of early compartment syndrome.

Following a diagnosis of early compartment syndrome in the patient, an emergency right lower extremity four-compartment fasciotomy was planned and the operating room (OR) team was called in. A multi-planar knee-spanning external fixator system was placed for soft tissue and bone stabilization. The patient returned to the OR on day 3 and 4 for repeat irrigation and debridement of the fasciotomies, as well as removal of the external fixator and open reduction internal fixation of the patient’s tibial plateau fracture and fasciotomy closure. Antibiotics were continued for 24 hours following the completion of the patient’s fasciotomies. The patient remained in the hospital for ten days improved pain control and rehabilitation. He was discharged home with home health care.

## Discussion

It is known that the most common cause of lipohemarthrosis is traumatic knee injury, with lipohemarthrosis even being considered pathognomonic for acute fracture [1]. Considering the fact that knee traumas account for hundreds of thousands of ED visits per year, it is necessary that clinicians maintain a high index of suspicion for lipohemarthrosis when patients present with knee traumas or intra-articular fractures. This urgency is only compounded by the additional risk of compartment syndrome that may accompany lipohemarthrosis. The extrusion of fat and blood from the fracture causes pressure build-up within the joint space, leading to increased compartment pressures within the joint [9]. As pressure increases within the closed fascial space, blood flow, and tissue perfusion reduce, leading to nerve and muscle dysfunction, extremity deformities, paralysis, and in some cases, amputation if the compartment syndrome is left untreated [10,11]. Surgical interventions like compartment fasciotomies may also be required. Therefore, due to the grave consequences of untreated compartment syndrome, it is of utmost importance that clinicians are able to recognize lipohemarthrosis and its potential resultant compartment syndrome in patients. 

X-rays may allow for the detection of lipohemarthrosis; however, they are less sensitive than other imaging modalities [1]. Because of this, CTs are considered the gold standard for lipohemarthrosis visualization because they allow for easier identification of the tell-tale fat-fluid levels formed when fat and blood from the fracture separate within the joint space [1]. While MRIs are also helpful for the identification of fat-fluid levels, the decreased availability and increased price of this form of imaging make it less desirable, and thus CTs without contrast are considered sufficient when attempting to visualize lipohemarthrosis. 

The particular case described in this paper is unique in that the patient sustained a right proximal intra-articular tibial fracture, right knee effusion, right proximal fibular fracture, and developed lipohemarthrosis after falling only 3 feet during a workplace accident. While landing on his right lower extremity in varus stress likely played a part in the severity of his injuries, the patient’s daily smoking habits are also notable when exploring the traumatic nature of his injury. Research demonstrates that tobacco smoking has a negative effect on bone turnover and may cause lower bone mass, leading to increased risk of fracture, osteoporosis, and certain skeletal system disorders [12]. 

Another notable aspect of this particular case is its clear delineation of the onset of compartment syndrome. Though the patient did not show signs of compartment syndrome upon admission to the ED, and though the first, second, and third re-evaluations of the patient did not yield any signs or symptoms of compartment syndrome, upon the fourth re-evaluation, his right lower extremity was warm and firm to touch, and his compartment pressure was measured at 62 mmHg. The onset of this early compartment syndrome took only 1 hour and 45 minutes following time of admittance to the ED. This case demonstrates that continual neurovascular and pressure checks of patients diagnosed with lipohemarthrosis are necessary, especially since onset of compartment syndrome can occur fairly rapidly and requires urgent intervention (eg. compartment fasciotomy) following diagnosis. 

## Conclusions

When treating patients presenting with knee trauma or intra-articular fractures, it is important that clinicians maintain a high index of suspicion for lipohemarthrosis and onset of early compartment syndrome. X-ray and CT imaging allows for visualization of intra-articular fractures, as well as the classic fat-fluid levels that indicate lipohemarthrosis. Frequent re-evaluations of patients diagnosed with lipohemarthrosis are necessary, especially since the resultant fluid and pressure build-up within joint spaces may lead to the development of compartment syndrome in patients. Checking compartment pressures with a manometer allows for the monitoring of compartment syndrome progression in order to ensure that surgical intervention via compartment fasciotomy occurs in a timely manner if indicated. 
